# Early diagnosis of pancreatic cancer: neutrophil gelatinase-associated lipocalin as a marker of pancreatic intraepithelial neoplasia

**DOI:** 10.1038/sj.bjc.6604329

**Published:** 2008-04-08

**Authors:** N Moniaux, S Chakraborty, M Yalniz, J Gonzalez, V K Shostrom, J Standop, S M Lele, M Ouellette, P M Pour, A R Sasson, R E Brand, M A Hollingsworth, M Jain, S K Batra

**Affiliations:** 1 Department of Biochemistry and Molecular Biology, University of Nebraska Medical Center, Omaha, NE 68198, USA; 2 Eppley Institute for Cancer Research, University of Nebraska Medical Center, Omaha, NE 68198, USA; 3 Department of Societal and Preventive Medicine, University of Nebraska Medical Center, Omaha, NE 68198, USA; 4 Department of Pathology and Microbiology, University of Nebraska Medical Center, Omaha, NE 68198, USA; 5 Department of Pathology and Microbiology, University of Nebraska Medical Center, Omaha, NE 68198, USA; 6 Department of Surgery, University of Nebraska Medical Center, Omaha, NE 68198, USA; 7 Division of Gastroenterology, University of Pittsburgh Medical Center, Pittsburgh, PA 15213, USA

**Keywords:** pancreatic cancer, diagnosis, PanIN, lipocalin, NGAL

## Abstract

Pancreatic cancer is a highly lethal malignancy with a dismal 5-year survival of less than 5%. The scarcity of early biomarkers has considerably hindered our ability to launch preventive measures for this malignancy in a timely manner. Neutrophil gelatinase-associated lipocalin (NGAL), a 24-kDa glycoprotein, was reported to be upregulated nearly 27-fold in pancreatic cancer cells compared to normal ductal cells in a microarray analysis. Given the need for biomarkers in the early diagnosis of pancreatic cancer, we investigated the expression of NGAL in tissues with the objective of examining if NGAL immunostaining could be used to identify foci of pancreatic intraepithelial neoplasia, premalignant lesions preceding invasive cancer. To examine a possible correlation between NGAL expression and the degree of differentiation, we also analysed NGAL levels in pancreatic cancer cell lines with varying grades of differentiation. Although NGAL expression was strongly upregulated in pancreatic cancer, and moderately in pancreatitis, only a weak expression could be detected in the healthy pancreas. The average composite score for adenocarcinoma (4.26±2.44) was significantly higher than that for the normal pancreas (1.0) or pancreatitis (1.0) (*P*<0.0001). Further, although both well- and moderately differentiated pancreatic cancer were positive for NGAL, poorly differentiated adenocarcinoma was uniformly negative. Importantly, NGAL expression was detected as early as the PanIN-1 stage, suggesting that it could be a marker of the earliest premalignant changes in the pancreas. Further, we examined NGAL levels in serum samples. Serum NGAL levels were above the cutoff for healthy individuals in 94% of pancreatic cancer and 62.5% each of acute and chronic pancreatitis samples. However, the difference between NGAL levels in pancreatitis and pancreatic cancer was not significant. A ROC curve analysis revealed that ELISA for NGAL is fairly accurate in distinguishing pancreatic cancer from non-cancer cases (area under curve=0.75). In conclusion, NGAL is highly expressed in early dysplastic lesions in the pancreas, suggesting a possible role as an early diagnostic marker for pancreatic cancer. Further, serum NGAL measurement could be investigated as a possible biomarker in pancreatitis and pancreatic adenocarcinoma.

Pancreatic adenocarcinoma is one of the most aggressive cancers, with an estimated 37 000 new cases and 34 000 deaths in 2007 in the United States alone (Jemal *et al*, 2007). The poor prognosis is to a large extent due to the fact that only 15–20% of pancreatic adenocarcinoma cases are resectable, and a mere 5% of patients survive 5 years (Cleary *et al*, 2004). The current treatment modalities for this malignancy, surgery, radiation, and chemotherapy, alone or in combination, have done little to improve the grim outlook; and once metastasis has set in, death is almost certain (Li *et al*, 2004). Nevertheless, an understanding of the pathogenesis of pancreatic adenocarcinoma, along with the development of new treatment methods and diagnostic techniques aimed at early detection, may facilitate effective prevention, and increase survival rates among patients.

Using microarray analysis, many laboratories have reported the differential expression of several novel genes associated with the progression of pancreatic cancer (Argani *et al*, 2001; Han *et al*, 2002; Terris *et al*, 2002). Of these genes, we chose to focus our interest on *NGAL* (neutrophil gelatinase-associated lipocalin), which is known to be overexpressed in pancreatic cancer (Han *et al*, 2002).

Neutrophil gelatinase-associated lipocalin is also known variously as NRL (neu-related lipocalin), oncogene 24p3, uterocalin, and lipocalin 2 (LCN2). It is a 24-kDa glycoprotein stored in specific granules of human neutrophils (Cowland and Borregaard, 1997). It belongs to the lipocalin family that comprises more than 50 known members, all of which are characterised by their low molecular weight and their ability to bind to and transport small lipophilic substances (Bratt, 2000; Xu and Venge, 2000). Although its structure is known, the specific functions of NGAL are yet to be elucidated. It is expressed during the transit of granulocytes in the bone marrow, which suggests a possible role for it in granulocyte maturation (Borregaard *et al*, 1995). More recently, NGAL has been shown to participate in iron trafficking (Yang *et al*, 2003) and proposed to participate in the iron depletion strategy used by the innate immune system against bacterial pathogens (Goetz *et al*, 2002). By regulating the delivery of iron into cells, it has been demonstrated to regulate iron-responsive genes that are important in the differentiation of primordial cells (Mori *et al*, 2003). Further, upregulation of NGAL in haematopoietic stem cells subjected to IL-3 starvation has been shown to promote apoptosis (Jayaraman *et al*, 2005). Thus, NGAL has a multifaceted role in several key cellular functions.

The deregulated expression of NGAL has been reported in several benign and malignant conditions. Neutrophil gelatinase-associated lipocalin expression was significantly upregulated in ovarian cancer tissues with borderline and grade I tumours showing the highest expression (Lim *et al*, 2007). Its expression was also increased in oesophageal dysplasia and squamous cell carcinoma, suggesting that it could be a marker of early dysplastic change (Zhang *et al*, 2007). Neutrophil gelatinase-associated lipocalin–matrix metalloproteinase-9 (MMP-9) complexes were identified in nearly 84% of urine sample from breast cancer patients while being absent from matched control samples (Fernandez *et al*, 2005). Neutrophil gelatinase-associated lipocalin was also identified as an independent indicator of poor prognosis in these patients (Bauer *et al*, 2007). Thus, NGAL appears to be a marker of dysplasia and thus could be useful to identify such changes in high-risk patients.

The idea of a possible link between NGAL and pancreatic cancer was first presented by Furutani *et al* (1998), who identified NGAL as one of the genes significantly overexpressed in pancreatic cancer cell lines. This observation was confirmed by Argani *et al* (2001). Three molecules, TFF2 (trefoil factor 2), PSCA (prostate stem cell antigen), and NGAL, were scored very highly in tumours compared to the normal pancreas. Finally, in 2002, two independent groups highlighted the possible role of NGAL in pancreatic cancer. The first group comprising Han *et al* (2002) used high-density cDNA microarray performed on neoplastic *vs* normal pancreatic cells and observed a 27-fold upregulation of NGAL in three pancreatic cancer cell lines compared to the normal pancreas. Following this, Terris *et al* (2002) identified NGAL in a search for markers of IPMN (intraductal papillary mucinous neoplasm), a precursor lesion known to lead to invasive carcinoma. More recently, Iacobuzio-Donahue *et al* (2003a, 2003b), exploring the global gene expression pattern in pancreatic adenocarcinoma using cDNA microarrays, reported a significant overexpression of NGAL in pancreatic cancer. However, the association of NGAL expression with the progression of pancreatic cancer and the possible role of plasma/serum NGAL levels as a diagnostic/prognostic marker in this lethal malignancy has not yet been explored.

Our laboratory is working for the past decade in trying to uncover the molecular and cellular mechanisms that drive the tumorogenic and metastatic potential of pancreatic cancer cells. Given the reported overexpression of NGAL in pancreatic cancer cell lines and its possible role in tumour cell differentiation, apoptosis, and inflammation, we explored the hypothesis that NGAL plays an important role in the early stages of pancreatic cancer pathogenesis and that its detection could potentially be useful in the diagnosis of pancreatic cancer. The expression of NGAL in pancreatic intraepithelial neoplasia (PanIN) lesions of various grades and in foci of pancreatitis adjacent to the areas of adenocarcinoma was examined by immunohistochemistry. We also examined NGAL transcript and protein expression in pancreatic cancer cell lines with varying degrees of differentiation to evaluate if NGAL expression correlates with epithelial differentiation. Further, we analysed NGAL levels in serum of patients with pancreatic cancer to investigate whether serum NGAL could be used to distinguish patients with pancreatic cancer from those with pancreatitis or disease-free pancreas.

## MATERIALS AND METHODS

### Tissue samples and cell lines

Eight pancreatic tissue samples from healthy donors, 1 from acute, 3 from chronic pancreatitis, and 27 from PDAC (pancreatic ductal adenocarcinoma) patients were collected after prior consent and fixed in formalin. In addition, 2 normal pancreatic tissue samples from healthy donors, 8 chronic pancreatitis, and 14 pancreatic adenocarcinoma samples were obtained at the time of primary surgery after obtaining appropriate consent and snap-frozen in the liquid nitrogen for RNA analysis. Samples were collected under a protocol approved by the Institutional Review Board at the University of Nebraska Medical Center (Andrianifahanana *et al*, 2001). Thirteen pancreatic adenocarcinoma cell lines with different levels of differentiation (poorly, moderately, and well differentiated) (Sipos *et al*, 2003) were examined: MiaPaCa, Panc1, Panc89, QGP1, S2CP9, AsPC1, BxPC3, Capan1, HPAF, HPAC, Colo357, Hs766T, and SW1990.

### Blood samples and isolation of serum

Blood samples were obtained from in-patients at the University of Nebraska Medical Center (UNMC, Omaha, NE, USA) under a research protocol approved by the local institutional review board (IRB's 209-00-EP). Whole blood was transferred from an anticoagulant citrate dextrose solution formula A-capped tube (ACD-A) to a conical tube (Nalgene, Rochester, NY, USA). Samples were centrifuged for 10 min at 1000 **g** in a swing bucket rotor at 4°C, and the serum was stored in cryovials at −80°C.

### Immunohistochemistry

Paraffin sections were deparaffinised in xylene, followed by rehydration in graded ethanol and treated for 20 min with 0.3%. H_2_O_2_/methanol to block endogenous peroxidase. Epitope retrieval was performed by microwaving the slides in 0.01 M citrate buffer (pH 6.0). The sections were blocked with normal goat serum for 1 h, followed by incubation with rabbit anti-NGAL pAb (1 : 1000 dilution) overnight at 4°C. The slides were then washed with PBS-T (3 × 5 min) before incubation with secondary antibody for 30 min. Slides were washed again (3 × 5 min) with PBS-T before incubating with the ABC solution. The reaction colour was developed by incubating the sections with DAB reagent. The slides were washed with distilled water, counterstained with haematoxylin, and dehydrated and mounted with Permount permanent mounting media (Fisher Scientific, Fair Lawn, NJ, USA).

All slides were observed under a Nikon Light Microscope and representative photographs taken. The intensity of immunoreactivity of NGAL was scored. The staining intensity was graded on a scale of 0 to 3+ (0 for no staining, 1+ for weak immunoreactivity; 2+ for moderate immunoreactivity; and 3+ for strong immunoreactivity). The percentage of cells that showed positive NGAL staining within the normal/cancerous region of a section was scored as follows: (1) 0–25% of cells positive; (2) 26–50%; (3) 51–75%; and (4) 76–100% cells positive for NGAL immunostaining. The staining intensity score and the percent immunoreactivity score were then multiplied to obtain a composite score. The values of the composite score ranged from a minimum of 0 to a maximum of 12. The Mann–Whitney *U*-test was used to compare the composite score among the groups (normal pancreas/pancreatitis/pancreatic adenocarcinoma). No multivariate analysis was carried out owing to the moderate number of events. Differences between the groups were defined as being statistically significant if the *P-*value was less than 0.05.

### RNA isolation and reverse transcription–PCR analysis

Total RNA from tissue samples and cell lines was isolated by guanidine isothiocyanate cesium chloride ultracentrifugation method. A total of 2 *μ*g RNA was reverse-transcribed using the SuperScript™ II RNase-Reverse Transcriptase System (Invitrogen, Carlsbad, CA, USA). The cDNA was then subjected to PCR with 1.5 mM magnesium chloride, 2.5 U Taq polymerase (Fermentas, Hanover, MD, USA) in a total volume of 50 *μ*l and specific primers for NGAL/LCN2. After 3 min initial denaturation at 94°C, 30 cycles of amplification (45 s 94°C, 45 s 60°C, 60 s 72°C) were performed. RPL13A was used as an internal control. The PCR products were electrophoretically resolved on 1% agarose gels stained with ethidium bromide. Photographs were taken under UV light, using the GelExpert software system (Nucleotech, San Mateo, CA, USA). The sequence of the forward primer for NGAL is 5′-TTCCTCGGCCCTGAATCATG-3′ and that of reverse primer is 5′-CTGGCGGCACCTGTGCACTCA-3′. RPL13A primers used were those reported previously (Andrianifahanana *et al*, 2001).

### Cell lysates and Western blot

Cells were lysed in modified RIPA buffer (50 mM Tris-HCl, pH 7.4; 0.25% Na-deoxycholate; 150 mM NaCl; 1% NP-40; 1 mM EDTA), supplemented with 1 mg ml^−1^ aprotinin, 1 mg ml^−1^ leupeptin, 5 mM NaF, 5 mM Na_3_VO_4_, and 1 mM phenylmethylsulphonyl fluoride. Total cellular protein was extracted on ice for 30 min and further cleared by centrifugation at 16 000 **g** for 10 min. The supernatant (comprising the protein extracts) was collected and stored at −80°C until further use.

The total protein was electrophoretically fractionated on 15% Laemmli SDS–polyacrylamide gels and electroblotted onto PVDF membranes. Blotted membranes were blocked in 5% BSA and subsequently exposed to primary antibodies specific for NGAL, diluted (1 : 1000) in PBS. The rabbit polyclonal anti-NGAL antibody, generously provided by Dr Gould (University of Wisconsin, Madison, WI), was raised against recombinant NGAL protein (Stoesz *et al*, 1998). After incubation with the appropriate secondary antibody, the membranes were treated with ECL reagent (Amersham Biosciences, Piscataway, NJ, USA) and exposed to autoradiographic films. The membranes were then stripped and re-probed with antibodies specific for *β*-actin.

### Determination of serum NGAL levels by sandwich ELISA

The concentration of NGAL in the serum of patients was determined by using the NGAL Rapid ELISA Kit (Antibody Shop, Gentofte, Denmark). Serum from patients with acute or chronic pancreatitis (*n*=8 in each group) and pancreatic cancer (*n*=16) were tested. Serum from healthy donors (*n*=8) were chosen as the control. Each sample was diluted 100-fold using the sample diluent provided with the kit. A volume of 50 *μ*l horseradish peroxidase-conjugated detection antibody was added to each well of a 96-well plate precoated with NGAL antibody. Thereafter, 50 *μ*l each of the calibrator (provided by the manufacturer), sample (pancreatitis, pancreatic cancer, or healthy control serum sample), or sample diluent alone (blank) was rapidly added to the corresponding wells. After incubating at room temperature for 30 min with agitation (200 min^−1^), the contents of the microwell were aspirated and replaced with 100 *μ*l of the chromogenic peroxidase substrate tetramethylbenzidene (TMB). The plate was incubated for 15 min in the dark, the reaction stopped by adding a ‘stop solution’, and the absorbance read at 450 nm with a microplate reader.

## RESULTS

### Immunohistochemistry of pancreatic tumours

**Figure 1:**
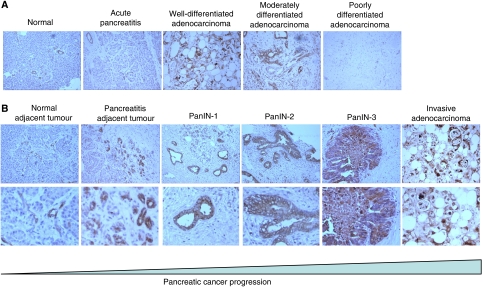
A representative immunohistochemical analysis of NGAL during the pathogenesis of the pancreatic gland. Representative sections of formalin-fixed, paraffin-embedded tissues immunolabelled with polyclonal anti-NGAL antibody using the horseradish peroxidase detection method with DAB substrate. Brown-coloured product indicates positive immunoreactivity. (**A**) Neutrophil gelatinase-associated lipocalin expression in normal pancreas, acute pancreatitis, and well-, moderately, and poorly differentiated pancreatic adenocarcinoma. The normal and pancreatitis tissue samples presented a weak staining in the small and small/medium size ducts, respectively. For pancreatic adenocarcinoma, a gradient of NGAL expression was detected from strongest in well-differentiated tumours to absent staining in poorly differentiated tumours. (**B**) Correlation of NGAL expression with pancreatic cancer progression. Neutrophil gelatinase-associated lipocalin positivity was identified in the premalignant PanIN lesions as early as PanIN-1. Pancreatitis surrounding the cancer stained as strongly as the PanIN areas. The staining intensity was weaker in the well-differentiated adenocarcinoma (top panel: original magnification × 10 and bottom panel: original magnification × 20). All sections were counterstained with haematoxylin.

To investigate the association between NGAL expression and pancreatic cancer pathogenesis, the NGAL expression pattern in the pancreas was investigated either by immunohistochemistry or RT–PCR. Immunohistochemistry experiments were performed on 39 formalin-fixed tissue samples representing normal (*n*=8), pancreatitis (*n*=4), and pancreatic adenocarcinoma (*n*=27), using the rabbit anti-NGAL polyclonal antibody (Stoesz *et al*, 1998). The results are summarised in the Tables 1, 2 and 3. In the eight sections of normal pancreas investigated, a weak NGAL staining was detected, restricted to the small ducts (Figure 1). A weak staining was also detected in some medium size ducts (average composite score: 1). The staining appeared cytoplasmic for the small ducts, and luminal for the larger ducts. In addition, a subpopulation of cells within the islets also appeared positive for NGAL (1–2 cells per islet). One acute and three chronic pancreatitis tissue sections were also examined. Neutrophil gelatinase-associated lipocalin antibody moderately labeled the small and medium size ducts (average composite score: 1), and in one case of chronic pancreatitis, the centroacinar cells (Figure 1). Thus, a weak and restrictive pattern of NGAL expression was observed in all non-malignant cells.

All the 27 pancreatic adenocarcinoma tissue sections were positive for NGAL expression, although with a heterogeneous pattern of staining (Table 3). Most (80–90%) of the medium and larger sized ducts were labeled with the antibody. However, a range of staining patterns was observed in the foci of pancreatitis and PanIN surrounding the adenocarcinoma areas. Although the foci of pancreatitis directly surrounding the cancer were strongly positive for NGAL (Figure 1), a strong staining was noted in all the PanIN lesions (Table 2), with an intense staining observable as early as PanIN-1 (Figure 1). Pancreatic intraepithelial neoplasia-1 lesions had an average composite score of 11.4, PanIN-2 of 11.25, and the PanIN-3 of 9.88 (Table 2). There was a statistically significance difference (*P*<0.004.) between the three grades of PanIN lesions (*P*<0.004). In well-established adenocarcinoma, however, the NGAL staining appeared less intense (Figure 1), and in some cases, negative. The intensity of the staining and the number of positive cells were directly associated with the degree of differentiation, with well-differentiated cancer staining more than the poorly differentiated areas (Figure 1). The mean composite score was 4.23 for well-differentiated pancreatic adenocarcinoma and 3.33 for the moderately differentiated pancreatic adenocarcinoma (Table 3). All the poorly differentiated adenocarcinomas were uniformly negative. The normal pancreas adjacent to the malignant area presented a labeling pattern identical to that observed in the normal pancreas of healthy individuals with immunostaining restricted to the small ducts. A few strongly positive cells were also observed in the islets, localised to within the close proximity of the tumour tissue. A significant difference was observed in NGAL expression (as measured by the composite score) between the normal and cancerous areas (*P*<0.0001). Thus, whereas sections of the normal pancreas and pancreatitis (in the absence of associated malignancy) expressed NGAL weakly, high levels of expression were noted in the areas of PanIN, with comparable levels in foci of pancreatitis surrounding the cancer. Neutrophil gelatinase-associated lipocalin staining in invasive adenocarcinoma, however, directly varied with the degree of differentiation with well differentiated the most intense and poorly differentiated the least.

### RT–PCR of tissues and cell lines

**Figure 2:**
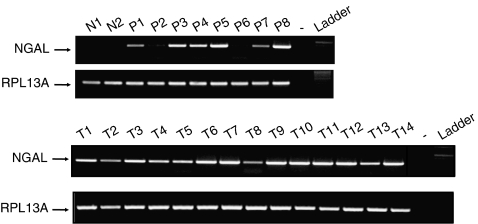
Neutrophil gelatinase-associated lipocalin transcript expression in normal, pancreatitis, and pancreatic cancer tissues. Neutrophil gelatinase-associated lipocalin expression was investigated by RT–PCR on 2 normal pancreas (N1 and N2), 8 chronic pancreatitis (P1–P8), and 14 pancreatic cancer tissue samples (T1–T14), respectively. RPL13A expression was used as an internal reference.

**Figure 3:**
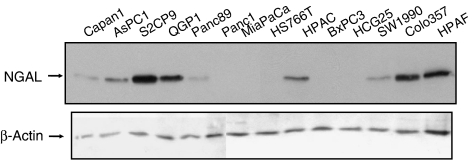
Western blot analysis of NGAL in a panel of pancreatic cancer cell lines. Protein lysates from 14 pancreatic cancer cell lines were resolved on a 15% SDS–PAGE gel and transferred to a PVDF membrane. After blocking with 5% BSA, the blot was hybridised with anti-NGAL antibodies (upper panel), stripped and re-probed with anti-*β*-actin (lower panel) to demonstrate loading of proteins in all lanes.

Having examined NGAL expression in tissues, we then investigated the variation at the transcript level both in tissues and cell lines. An RT–PCR analysis of pancreatic tissue samples (2 normal pancreas, 8 pancreatitis, and 14 pancreatic cancer) revealed a faint amplification product (confirmed as *NGAL* after sequencing) in the normal tissues, a moderate-to-strong band in pancreatitis, and a strong amplification product in all pancreatic cancer samples (Figure 2). Neutrophil gelatinase-associated lipocalin expression was also investigated in pancreatic cancer cell lines by either RT–PCR (data not shown) or Western blot. Western blot revealed a high level of expression of the 24 kDa NGAL protein in all the well-differentiated pancreatic cancer cell lines (S2CP9, Colo357, HPAF, and HPAC), weak expression in the moderately differentiated pancreatic cancer cell lines (Capan-1, SW1990, HS766T, ASPC-1, and Panc89), and no detectable expression in the poorly differentiated pancreatic cancer cell lines (Panc1, MiaPaCa, BxPC3, and HCG25) (Figure 3). The rabbit polyclonal antibody against *β*-actin JLA20 (Sigma-Aldrich, St Louis, MO, USA) was used to control the equity in sample loading. Pancreatic ductal cancer cell lines are classified as well, moderately, or poorly differentiated according to their ultrastructural features, including the integrity of membrane-bound structures, presence of mucin granules, cell organelles, nuclear and cellular polymorphism, cell polarity, and lumen formation (Sipos *et al*, 2003). These results suggest a differential overexpression of NGAL in well- and moderately differentiated pancreatic cancer cell lines, thereby suggesting a potential association between NGAL expression and the differentiation of pancreatic cancer cells.

### Quantitative ELISA for NGAL levels in serum

**Figure 4:**
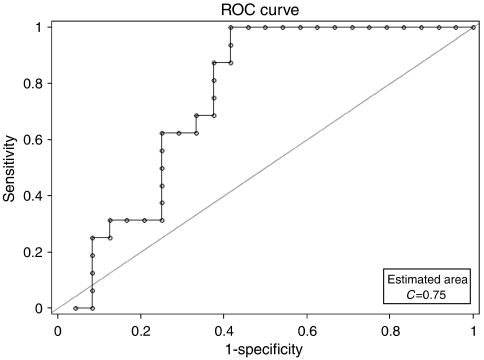
Receiver operating characteristic (ROC) curve analysis of diagnostic sensitivity and specificity of the plasma NGAL ELISA assay. The performance of plasma NGAL measurement assay in discriminating patients with pancreatic cancer from those without cancer (normal, acute, and chronic pancreatitis) was evaluated. The area under the curve (AUC) was 0.75, suggesting that the test is fairly accurate in distinguishing the two groups.

From the preceding experiments, we observed that NGAL was overexpressed in pancreatic adenocarcinoma, but very weakly in normal and pancreatitis tissues. Hence, to examine if quantitative analysis of NGAL levels in blood could distinguish pancreatic cancer from pancreatitis, we analysed serum samples for NGAL levels. Using an established cutoff for healthy individuals (106 ng ml^−1^), serum NGAL levels in 94% of the pancreatic cancer samples. A total of 62.5% of the acute and chronic pancreatitis serum samples each also had NGAL levels above the upper limit for normal. The mean and median NGAL levels are listed in Table 4. Further, the serum levels of NGAL in normal *vs* pancreatitis (acute/chronic) and pancreatic cancer samples were analysed by non-parametric tests (Table 5). The Kruskal–Wallis test showed a significant difference (*P*<0.002) in the distribution of serum NGAL levels across the groups (normal, acute/chronic pancreatitis, and pancreatic cancer). However, when a pair-wise comparison was performed using the Wilcoxon test, we observed that although serum NGAL levels were significantly higher in acute (*P*=0.035), chronic pancreatitis (*P*=0.035), and pancreatic cancer (*P*=0.004) compared to healthy controls, there was no significant difference between the pancreatitis and pancreatic cancer samples (*P*=1.000). This suggested that NGAL levels in the serum were elevated both in inflammatory and neoplastic pathologies of the pancreas. We also analysed the ability of the serum NGAL assay to distinguish pancreatic cancer from non-cancer cases by fitting the data to a simple logistic regression model where samples were categorised as ‘cancer’ or ‘no-cancer’, and mean NGAL was used as the predictor. The analysis revealed that serum NGAL levels in cancer were not significantly different from non-cancer cases (comprising normal and pancreatitis cases) to be diagnostically useful (*P*=0.101). However, a ROC curve analysis revealed that the area under the curve was 0.75 (Figure 4), which indicates that the test is fairly accurate in classifying cases as ‘cancer’ or ‘no-cancer’. Taken together, the results suggest that although the measurement of serum NGAL alone may not provide diagnostic accuracy in distinguishing pancreatic cancer patients from those with pancreatitis, it warrants further exploration as a diagnostic marker for pancreatic cancer in combination with other markers. Further, similar to its elevation in the early stages of acute renal injury, NGAL could be explored as an early marker of pancreatic damage in acute pancreatitis.

## DISCUSSION

NGAL is the human homologue of the murine molecule known as oncogene 24p3 (mouse) and neu/HER2-related lipocalin (rat). Oncogene 24p3 was first identified as being overexpressed in oncogene-mediated cell transformation (Hraba-Renevey *et al*, 1989). The protein was named NGAL because it was observed to form a complex with the gelatinase MMP9 (Kjeldsen *et al*, 1993). Neutrophil gelatinase-associated lipocalin is a multifunctional protein and is released from activated neutrophils (Elneihoum *et al*, 1996) and thought to act as a bacteriostatic agent (Goetz *et al*, 2002). Additionally, it also appears to be important in cellular differentiation being highly expressed in fetal hypertrophic chondrocytes, developing skeletal muscle fibers, and during myocardial development. The expression of NGAL is highest just before the onset of cellular differentiation (Zerega *et al*, 2000). Therefore, it is suggested that NGAL favours differentiation of primordial cells.

The observation that induction of NGAL correlates with apoptosis led to the hypothesis that NGAL is a pro-apoptotic factor (Huang *et al*, 1999; Ryon *et al*, 2002). This hypothesis was furthered strengthened by the observation that NGAL induced the apoptosis of neutrophils through an autocrine pathway (Devireddy *et al*, 2001). In a recent publication, Tong *et al* (2005) showed that although NGAL expression is closely associated with apoptosis, it also favours cell survival. Thus, NGAL appears to have a complex functional role.

Neutrophil gelatinase-associated lipocalin has been reported to be overexpressed in pancreatic cancer cell lines and tissues (Furutani *et al*, 1998; Argani *et al*, 2001; Han *et al*, 2002). However, its role in the early stages of pancreatic cancer pathogenesis remains to be elucidated. In this study, we observed a specific and differential expression pattern for NGAL in pancreatic adenocarcinoma compared to that in the normal. Neutrophil gelatinase-associated lipocalin expression was low in the normal pancreas and in pancreatitis, but very high in the early dysplastic lesions (PanINs), being detectable as early as PanIN-1; it has been previously explored as a marker of premalignant lesions in ovarian cancer (Lim *et al*, 2007). The intense staining noted in the early PanIN lesions suggests that NGAL could be a marker of early dysplasia in the pancreas. Pancreatic intraepithelial neoplasia lesions have been reported to be present in the ducts of histologically normal pancreatic tissue (Andea *et al*, 2003; Gupta and Mazzara, 2005; Pan and Wang, 2007), including a progressive increase in their frequency from normal pancreas through pancreatitis to pancreatic cancer (16, 60, and 82%, respectively) (Andea *et al*, 2003). This has been proposed as evidence to support the role of these lesions as harbingers of ductal adenocarcinoma. However, in our study, we did not observe any PanIN lesions in the sections of normal pancreas, possibly attributable to the small number of normal tissues examined. In the areas of adenocarcinoma itself, NGAL expression correlated positively with the grade of differentiation (moderate expression for the highly differentiated *vs* negative for the poorly differentiated cancer). As the degree of differentiation of pancreatic cancer correlates inversely with its aggressiveness, hence detection of NGAL in tissue sections appears to be a good marker of the early, less belligerent stages of this malignancy.

The early diagnosis of pancreatic cancer is difficult owing to the late presentation of symptoms. A study comparing various diagnostic tests used in pancreatic cancer revealed that imaging techniques are the most sensitive method to identify patients suspected to have pancreatic cancer (Go *et al*, 1981). However, most patients in this study had unresectable tumours when first identified. Hence, a marker to identify early stage cancer or high-grade dysplasia would be immensely useful to improve survival. Neutrophil gelatinase-associated lipocalin being upregulated in the earliest stage of pancreatic intraepithelial neoplasia could be useful as a marker of dysplasia in high-risk patients (e.g. long-standing chronic pancreatitis and hereditary pancreatitis). However, it is unlikely by itself to be sufficiently sensitive to detect all cases. Its performance in combination with other potential early diagnostic markers needs to further exploration.

Serum NGAL measurement has been shown in studies to be elevated early in acute renal injury (Mishra *et al*, 2005; Dent *et al*, 2007) and in patients with borderline tumours of the ovary (Lim *et al*, 2007). Having established the differential overexpression in pancreatic cancer, specifically in the PanIN lesions, we next investigated whether the measurement of NGAL levels in the serum could be useful to distinguish pancreatic cancer from non-cancer cases (including, pancreatitis). Although serum NGAL levels were significantly elevated both in inflammation (pancreatitis) and malignancy of the gland, there was no significant difference between the serum levels in pancreatitis *vs* that in pancreatic cancer. This suggests that serum NGAL level alone is inadequate in distinguishing pancreatic cancer from chronic or acute pancreatitis. The role of NGAL as a mediator of inflammation has been reported previously following the nearly 55-fold increase in the mucosal release of NGAL in patients with colitis and proctitis (Carlson *et al*, 2002). Studies examining the effect of cytokines on hepatocytes showed that IL-*β* but not IL-6 induced the release of NGAL, suggesting a differential regulation of NGAL secretion by inflammatory cytokines (Jayaraman *et al*, 2005). It has been suggested that the upregulation of NGAL in liver cells in response to cytokines is a negative feedback mechanism to counter the effects of IL-1*β*-induced inflammation. Analogously, NGAL overexpression could serve to counter tumour-induced inflammatory response in pancreatic cancer (Jayaraman *et al*, 2005). The NGAL serum ELISA test appears to be fairly accurate in distinguishing pancreatic cancer from non-pancreatic cancer cases (area under the ROC curve=0.75). This raises the possibility that it might be useful in the diagnosis of pancreatic cancer in combination with other, more specific markers. Further, its significantly high levels in both acute and chronic pancreatitis suggested that serum NGAL levels could also be explored as a possible diagnostic marker in pancreatitis in the appropriate clinical context.

In the tumour microenvironment, one of the earliest changes that precedes the onset of a metastatic phenotype is an epithelial–mesenchymal transition associated with loss of E-cadherin expression, morphologic changes, and an increase in cellular motility (for review see Thiery, 2003a, 2003b). Hanai *et al* (2005) recently showed that NGAL has the ability to reverse this epithelial-mesenchymal transition, thereby diminishing invasiveness of cancer cells. Our results show that in pancreatic cancer, NGAL is predominantly expressed in the early dysplastic lesions as well as in the well-differentiated carcinomas (preserve ductal architecture); with almost no expression in the poorly differentiated tumours (have a predominantly mesenchymal appearance). These results led us to hypothesise that loss of NGAL expression drives the progression of pancreatic ductal carcinoma from a well to poorly differentiated tumour. Further experiments will need to be performed to prove this hypothesis. However if true, the development of recombinant NGAL-based therapies might slow down this epithelial-to-mesenchymal transformation and thereby the progression of this lethal cancer.

Pancreatic cells, either ductal, islet, or acinar, have the property to transdifferentiate into any other pancreatic cell type. Neutrophil gelatinase-associated lipocalin might initiate this process in the pancreatic cells. The identification of the factor(s) that induce(s) NGAL overexpression in PanIN lesions as early as PanIN-1A is essential to understand the mechanisms underlying the initiation and regulation of pancreatic ductal cell transformation, and transdifferentiation. A control over transdifferentiation of pancreatic cells might provide a new therapeutic approach for pancreatic cancer and possibly a cure for diabetes mellitus.

## Figures and Tables

**Table 1:** Immunohistochemical assessment of NGAL expression in pancreatitis and PDAC

**Tissue specimen**	***N***	**Average composite score**
Normal pancreas	8	1±0
Pancreatitis	4	1±0
Adenocarcinoma	27	4.26±2.44^a^

*N*=specimen number; NGAL=neutrophil gelatinase-associated lipocalin; PDAC=pancreatic ductal adenocarcinoma.

The staining intensity was graded on a scale of 0–3 (0=no staining, 1=weak, 2=moderate, and 3=strong staining). The percentage of cells that were positive for NGAL were scored as follows: 1=<25% cells positive, 2=25–50% cells positive, 3=50–75% cells positive, and 4=75–100% cells positive. The staining intensity score and percent positivity score for each section were multiplied to obtain a composite score for that section. The composite score ranged from 0 to 12.

a The difference in the staining score between normal and adenocarcinoma of the pancreas was statistically significant: *P*<0.0001.

**Table 2:** NGAL expression in pancreatic intraepithelial neoplasia by immunohistochemistry

**PanIN**					
**PanIN-1**		**PanIN-2**		**PanIN-3**	
*N* a	Average composite score	*N*	Average composite score	*N*	Average composite score
20	11.4±1.23	16	11.25±1.34	17	9.88±1.44^b^

NGAL=neutrophil gelatinase-associated lipocalin; PanIN=pancreatic intraepithelial neoplasia.

NGAL expression in the PanIN lesions adjacent to the areas of pancreatic cancer was calculated. The average composite score for PanIN lesions of a given grade was then calculated.

a *N* represents the number of tissue specimens containing PanIN lesions of a given grade.

b The difference in the composite score between PanIN-1, -2, and -3 stages was statistically significant: *P*<0.004.

**Table 3:** NGAL staining by immunohistochemistry in PC tissue sections

	**Well-differentiated**		**Moderately differentiated**		**Poorly differentiated**	
**Sample number**	**% positive cells (intensity of the staining)**	**Composite score**	**% positive cells (intensity of the staining)**	**Composite score**	**% positive cells (intensity of the staining)**	**Composite score**
1			25 (2)	2		
2	75 (3)	9				
3			25 (1)	1	75 (0)	0
4			75 (2)	6		
5	25 (2)	2			75 (0)	0
6	25 (2)	2			75 (0)	0
7			25 (2)	2		
8			50 (3)	6		
9	50 (3)	6	25 (2)	2		
10	25 (3)	3	50 (2)	4		
11			25 (3)	3		
12			50 (2)	4		
13			25 (1)	1		
14			25 (3)	3	75 (0)	0
15			75 (3)	9	25 (0)	0
16	50 (3)	6				
17	50 (3)	6				
18	50 (3)	6				
19	25 (3)	3			75 (0)	0
20	25 (3)	3			75 (0)	0
21	25 (3)	3			75 (0)	0
22	25 (3)	3	50 (2)	4		
23	25 (3)	3	25 (3)	3	50 (0)	0
24			25 (2)	2	75 (0)	0
25			25 (3)	3	75 (0)	0
26			25 (3)	3		
27			25 (2)	2		
Mean composite score		4.23±2.12		3.3±2		0

NGAL=neutrophil gelatinase-associated lipocalin; PC=pancreatic cancer.

A total of 27 PC tissue sections were investigated for NGAL expression by immunohistochemistry. The table presents the distribution of the composite scores in well-differentiated vs moderately and poorly differentiated cancer areas. The columns on the extreme right and at the bottom of the table represent the total composite score for a given PC section and the mean composite score for a given grade of differentiation, respectively. The scores were calculated as described in Table 1.

**Table 4:** Descriptive statistics of NGAL levels in serum

**Group**	**Diagnosis**	***N* a**	**Mean (ng ml^−1^)**	**Median (ng ml^−1^)**
I	Normal	8	44.19	43.43
II	Chronic pancreatitis	8	166.64	125.43
III	Acute pancreatitis	8	123.38	122.23
IV	Pancreatic cancer	16	151.65	143.75

NGAL=neutrophil gelatinase-associated lipocalin.

Serum NGAL levels were determined by a sandwich ELISA method. Mean and median NGAL levels were calculated for each of the four groups.

a *N* refers to the number of samples in a given group.

**Table 5:** Assessment of discriminative ability of the serum NGAL ELISA assay to distinguish normal patients from pancreatitis or pancreatic adenocarcinoma cases

**Comparison**	***P*-value^a^**
Pancreatic cancer *vs* normal	0.0042
Pancreatic cancer *vs* chronic pancreatitis	1.0000
Pancreatic cancer *vs* acute pancreatitis	1.0000
Chronic pancreatitis *vs* normal	0.0354
Acute pancreatitis *vs* normal	0.0354
Chronic pancreatitis *vs* ncute pancreatitis	1.0000

NGAL=neutrophil gelatinase-associated lipocalin.

Differences in serum NGAL levels between the four groups (outlined in Table 4) were analysed by the Wilcoxon test. The Bonferroni correction for multiple comparisons was applied to the data. A difference in mean serum NGAL level between any two groups was considered significant if the *P*-value<0.05.

a *P*-values for pair-wise comparisons are from the Wilcoxon test. These *P*-values were adjusted using the Bonferroni adjustment for multiple comparisons.
